# Spontaneous Cooling Enables High‐Quality Perovskite Wafers for High‐Sensitivity X‐Ray Detectors with a Low‐Detection Limit

**DOI:** 10.1002/advs.202410303

**Published:** 2024-10-21

**Authors:** Wenyi Wu, Jianqiang Zhang, Ciyu Liu, Jiankai Zhang, Hoajie Lai, Zhongqiang Hu, Hai Zhou

**Affiliations:** ^1^ International School of Microelectronics Dongguan University of Technology Dongguan Guangdong 523808 P. R. China; ^2^ School of Electronic Science and Engineering Xi'an Jiaotong University Xi'an Shaanxi 710049 P. R. China

**Keywords:** perovskite wafers, spontaneous cooling strategy, X‐ray detection

## Abstract

Developing high‐quality perovskite wafers is essential for integrating perovskite technology throughout the chip industry chain. In this article, a spontaneous cooling strategy with a hot‐pressing technique is presented to develop high‐purity, wafer‐scale, pinhole‐free perovskite wafers with a reflective surface. This method can be extended to a variety of perovskite wafers, including organic–inorganic, 2D, and lead‐free perovskites. Besides, the size of the wafer with diameters of 10, 15, and 20 mm can be tailored by changing the mold. Furthermore, the mechanism of spontaneous cooling for improving the quality of perovskite wafers is revealed. Finally, the high‐quality lead‐free Cs_3_Cu_2_I_5_ perovskite wafers demonstrate excellent X‐ray detection performances with a high sensitivity of 3433.6 µC Gy_air_^−1^ cm^−2^ and a low detection limit of 33.17 nGy_air_ s^−1^. Moreover, the Cs_3_Cu_2_I_5_ wafers exhibit outstanding environmental and operational stability even without encapsulation. These research presents a spontaneous cooling strategy to achieve wafer‐scale, high‐quality perovskites with mirror‐like surfaces for X‐ray detection, paving the way for integrating perovskites into electronic and optoelectronic devices and promoting the practical application of perovskite X‐ray detectors.

## Graphical abstract

A spontaneous cooling strategy with a hot‐pressing technique is presented to develop high‐purity, wafer‐scale, pinhole‐free perovskite wafers with a reflective surface. This method can be extended to a variety of perovskite wafers, including organic–inorganic, 2D, and lead‐free perovskites. This high‐quality lead‐free Cs_3_Cu_2_I_5_ perovskite wafers demonstrate excellent X‐ray detection performances with a high sensitivity of 3433.6 µC Gy_air_^−1^ cm^−2^ and a low detection limit of 33.17 nGy_air_ s^−1^, both of which are the best for Cs_3_Cu_2_I_5_ X‐ray detectors and comparable to other Pb‐based perovskite X‐ray detectors.

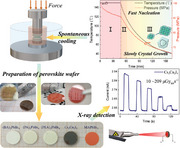

## 1 Introduction

In recent years, perovskites have attracted extensive attention as emerging semiconducting materials due to their outstanding photophysical properties, such as high light absorption coefficients, long carrier diffusion lengths, high and balanced electron–hole carrier mobilities, and high defect tolerance.^[^ ^1^, ^2^, ^3^, ^4^ ^]^ These merits make perovskite materials achieve great success, such as the efficiency of single‐cell solar cells based on perovskites exceeds 26%,^[^ ^5^ ^]^ and the external quantum efficiency (EQE) of perovskite LEDs is over 32%.^[^ ^6^ ^]^ Besides, perovskite semiconductors exhibit significant X‐ray absorption efficiency and the ability to generate a strong electrical signal from absorbed photons,^[^ ^7^, ^8^, ^9^, ^10^ ^]^ which is promising for direct X‐ray detection and imaging, particularly considering the exciting results in computed tomography (CT) imaging.^[^ ^9^ ^]^ However, to become the cornerstone of semiconductor architecture comparable to silicon, achieving perovskite‐based complementary metal oxide semiconductor (CMOS) chips is crucial.^[^ ^11^, ^12^, ^13^, ^14^ ^]^ There are currently many difficulties in implementing perovskite‐based CMOS chips, and we believe two main challenges are urgent: the first is to achieve p/n‐type of the perovskite, and the second is to prepare high‐quality perovskite wafers. Among these two major challenges, achieving high‐quality perovskite wafers is a prerequisite, which is the cornerstone of perovskite chips and a top priority for the perovskite to enter the entire chip industry chain. Therefore, exploring universal methods for the fabrication of high‐quality perovskite wafers is of great significance and value.

In this article, we demonstrate a spontaneous cooling strategy with a hot‐pressing technique to fabricate high‐purity, wafer‐scale, pinhole‐free perovskite wafers with a mirror‐like reflective surface, and the method can be expanded to various perovskite materials including organic–inorganic perovskite, 2D perovskite and lead‐free perovskite. Besides, various sizes of perovskites can also be obtained by our method, and the spontaneous cooling mechanism for improving the quality of perovskite wafers is revealed. Furthermore, these high‐quality perovskite wafers are applied for X‐ray detection with excellent performances. Under a 10 V bias, our lead‐free Cs_3_Cu_2_I_5_ perovskite wafer X‐ray detector shows sensitivity as large as 3433.6 µC Gy_air_^−1^ cm^−2^ with a detection limit lowered to 33.17 nGy_air_ s^−1^, both of which are the best for Cs_3_Cu_2_I_5_ X‐ray detectors and comparable to other Pb‐based perovskite X‐ray detectors. Our findings provide a spontaneous cooling strategy to achieve wafer‐scale, high‐purity, pinhole‐free perovskites with mirror‐like surfaces for X‐ray detection, which takes a big step toward achieving perovskite chips in electronics and optoelectronics.

## 2 Results and Discussion

**Figure 1:**
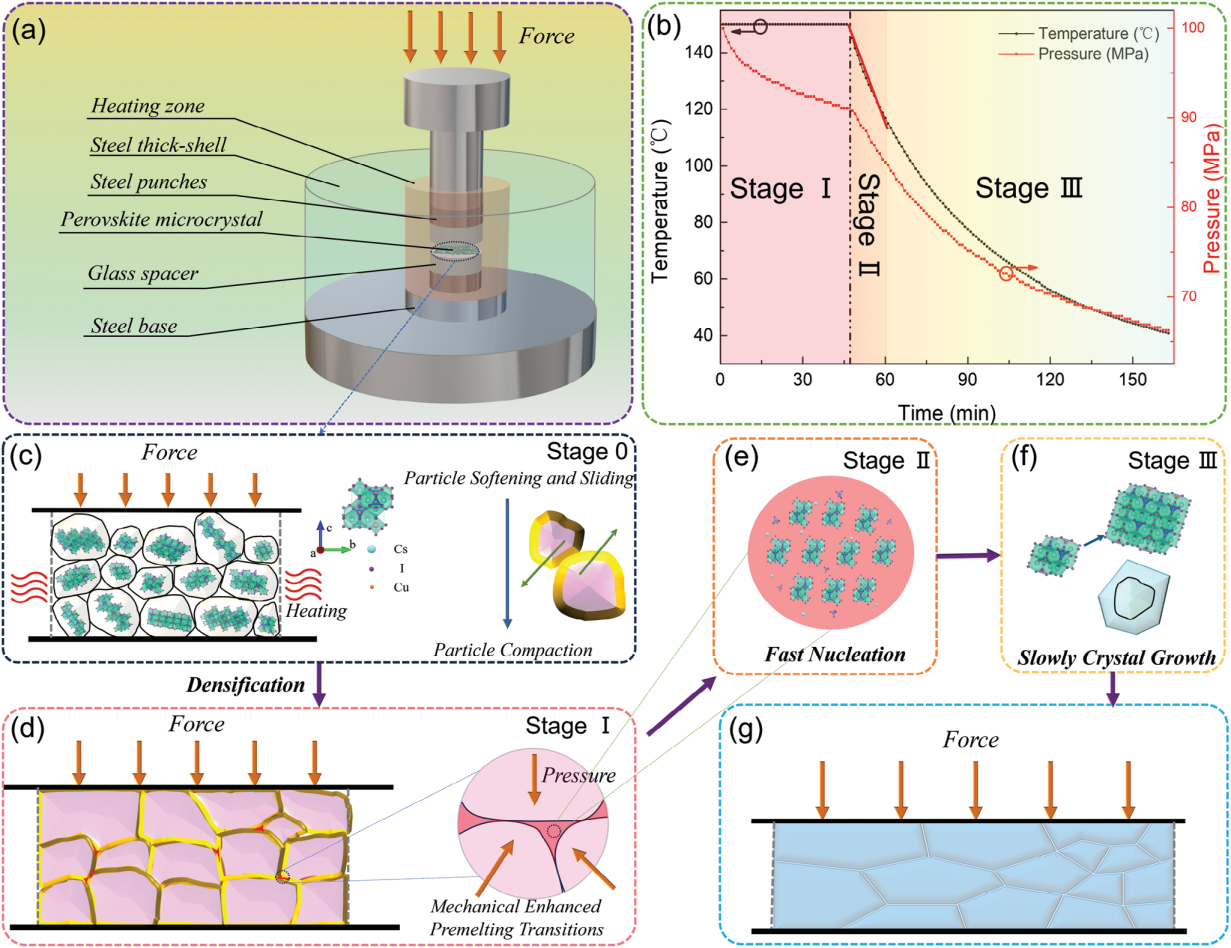
Spontaneous cooling mechanism for perovskite wafers in the hot‐pressing process. a) The experimental setup for spontaneous cooling. b) Temperature and pressure as a function of time during the spontaneous cooling process. c) Particle compaction, softening, and sliding. d) Densification and grain boundary pre‐melting transformation. e) Fast nucleation. f) Slow crystal growth. g) A prepared wafer with high densification, no pinholes, well‐defined large grains, and good grain boundary interconnection.

A hot‐pressing mold is used to achieve the preparation of a high‐quality perovskite wafer, which is illustrated in **Figure** **1**a. The mold is equipped with a heating mat. The temperature sensing and heating module is tightly attached to the pressure zone to obtain accurate real‐time temperature, the outer side is a solid stainless steel thick shell. We adopt the perovskite microcrystalline powders as the precursor, which have the advantage of being able to achieve controllable preparation of perovskite wafers at low cost through a solution method with high phase purity. Besides, numerous types of perovskite microcrystal powders can be prepared by a precipitation reaction with a non‐toxic antisolvent (such as diethyl carbonate), which will minimize the impact on the environment.

Figure 1b illustrates the real‐time temperature and pressure curves of the mold heating zone during the hot‐pressing and spontaneous cooling processes, which is composed of three main stages: isothermal heating stage (Stage I), rapid cooling stage (Stage II) and slow cooling stage (Stage III). In Stage I, the pressure gradually decreases after reaching the target pressure and drops more rapidly upon cessation of heating. The decrease in pressure can be attributed to grain sliding and deformation during densification, as well as mass transfer and pore filling.^[^ ^15^, ^16^, ^17^, ^18^, ^19^ ^]^ During the spontaneous cooling stage, the pressure decreases rapidly, owing to volume contraction resulting from thermal expansion and crystallization. We investigated the impact of varying quantities of microcrystalline powders (0.8 and 1.2 g) on pressure curves. As depicted in Figure S6a (Supporting Information), an increase in powder mass (1.2 g) will slightly accelerate the decrease in pressure. This effect can be attributed to enhanced elastic deformation under high‐pressure conditions, as well as more pronounced volume fluctuations caused by thermal expansion and contraction during spontaneous cooling processes associated with larger sample volumes. Furthermore, to depict the pressure behavior in wafers with different densities, we analyzed the pressure variation curves of Cs_3_Cu_2_I_5_ (wafer density of 4.292 g cm^−^^3^) and PEA_2_PbBr_4_ (wafer density of 2.215 g cm^−^^3^) with the same mass (0.8 g) during the hot‐pressing process, as illustrated in Figure S6b (Supporting Information). The finding reveals that PEA_2_PbBr_4_ wafers, possess a lower density and are inherently more resistant to compression. They exhibit more robust elastic deformation under saturation pressure, leading to a faster pressure drop before spontaneous cooling. Additionally, during the spontaneous cooling, the deceleration in pressure reduction for PEA_2_PbBr_4_ may be attributed to its lower thermal expansion coefficient relative to Cs_3_Cu_2_I_5_ wafers. Moreover, for the temperature curve of the spontaneous cooling stage, the initial cooling rate is high and diminishes over time. This cooling rate trend is attributed to the thermal insulation provided by the thick steel shell on the exterior, which promotes fast nucleation and slow high‐quality crystallization in this stage. In Stages II and III, the pressure and temperature show similar downward trends as the cooling time increases. The specific mechanisms of spontaneous cooling crystallization will be comprehensively discussed in subsequent sections.

For the perovskite wafers, the fabrication process mainly involves two steps: hot pressing densification and spontaneous cooling crystallization. The initial stage is the pressure transmission that occurs during compaction,^[^ ^15^ ^]^ which is named Stage 0, as shown in Figure 1c. The processes can be delineated by rearrangement and sliding of perovskite powders, complemented by their plastic and elastic deformation.^[^ ^16^ ^]^ Heating to soften perovskite grains prior to applying pressure is necessary, which facilitates particle rearrangement dynamics at low pressures. The uniaxial stress induces compaction, leading to the planarization of grains and the facilitation of grain boundary sliding.^[^ ^17^ ^]^ In this period, the pressure decreased over time, which made it necessary to regulate it manually to ensure it remained at the targeted pressure level. As the pressure escalates, the impact of plastic deformation intensifies, but the effect of elastic deformation ultimately prevails at even higher pressures.^[^ ^18^ ^]^ The relaxation time of pressure increases with the increase of pressure adjustment times, indicating that the compaction process seems to be saturated at high‐pressure levels.

When subjected to high stresses at a constant temperature, the pre‐melting transition starts and it will be preferentially from sharp edges or small particles, leading to a decrease in pressure (Stage I).^[^ ^19^ ^]^ Pre‐melting is a type of complexion transition that occurs at a crystalline surface or grain boundary, which refers to the formation of liquid‐like film at a temperature below the bulk melting temperature.^[^ ^20^, ^21^, ^22^ ^]^ Subsequently, the nanoparticles meet each other, small grains tend to disappear, and large grains tend to grow further, large grain growth is facilitated through the consumption of small grains. Grain growth could be driven by Ostwald ripening or particle migration and coalescence.^[^ ^23^, ^24^, ^25^, ^26^ ^]^

When the equipment cools down spontaneously, the grains continue to undergo growth and crystallization. The spontaneous cooling crystallization is further divided into two important steps: nucleation (in Figure 1e, Stage II) and growth (in Figure 1f, Stage III). Both steps require a decrement in the total Gibbs free energy, to attain the thermodynamic favorable.^[^ ^27^ ^]^ In the nucleation section, various nucleations did not occur simultaneously due to the different formation‐free energies, propagation from the initial stage to the critical nucleus necessitates a temporal interval. When the spherical nucleus radius *r* reaches a critical value *r^∗^*, the free energy ΔG* reaches maximum at an undercooling given by $\;\;\Delta G^{*}=16\pi \sigma^{3}T_{m}^{2}V_{S}^{2}/\left[3{\left(\Delta H\Delta T\right)}^{2}\right]$, where *Tm*, Vs, Δ*T* and Δ*H* are the melting temperature, volume of particles, indegree of undercooling, and enthalpy change, respectively. The size of particles is larger than *r^∗^*, grains grow stably, thereafter, ΔG* decreases rapidly. Correspondingly, the nucleation frequency (*I*) is given by $I\;=I_{0}\;\exp\left[-\Delta G^{*}/\left(kT\right)\right]$, where *I_0_* depends on *r ^∗^*, surface energy *σ* and the solution diffusion coefficient.^[^ ^28^, ^29^, ^30^ ^]^ The increase ΔT leads to decrease *r^∗^*, reduce ΔG*, and increase *I*. Given the correlation between the cooling rate, indegree of undercooling, and the frequency of nucleation, it becomes evident that an elevated cooling rate promotes nucleation at the interface. Thus, the higher cooling rate in the initial cooling stage facilitates a high density of homogeneous nuclei at the interface, which is beneficial for enhancing the quality of the crystal growth. As shown in Figure 1e, Stage II, under conditions of spontaneous cooling, the initial cooling rate is high, which ensures the fast nucleation of the pre‐melted area, resulting in a higher nucleation density and coverage at the grain boundaries, and suppresses the generation of harmful grain boundaries.

In the crystal growth section (Figure 1f), there is a dynamic process of disorder‐to‐order structural transformation at the liquid–solid interface, which can be described by an interfacial diffusion model.^[^ ^31^, ^32^ ^]^ The interface will move by a periodic potential field stemming from the lattice, and the interface energy will also change periodically. Crystal growth requires crossing the interfacial potential barrier, the driving force for crystallization dictates the quality of the interfacial growth, and the driving force is determined by the indegree of undercooling ΔT. Consequently, the correlation linking the rate of crystal growth to the driving force can be substituted with the rate and ΔT. In the absence of a potential barrier, the critical driving force ΔG*for continuous growth to occur can be formulated as Δ G* = πσ_0_ *g_m_*/*a* , the critical degree of undercooling ΔT* is described by:^[^ ^32^ ^]^

(1) $$\Delta \;T^{*}=\frac{\sigma_{0}g_{m}V_{m}T_{m}}{a\Delta \;H_{m}}$$

(2) $$g_{m}=\frac{1}{8}\pi^{4}n^{3}\exp\left(-\frac{1}{2}\pi^{2}n\right)$$

where *Tm*, Vs, and Δ*H* are the melting temperature, volume of particles, and enthalpy change, respectively. *a* is the atomic layer spacing in the direction perpendicular to the dense row surface, *n* is the number of atomic layers contained in the interface transition region, σ_0_ is the value of the interface energy, *g_m_* is the periodic relative amplitude of its interfacial energy, which changes with the interface position α. At a specified value of ΔT, the crystal growth rate is denoted by $R=\mu e^{-\frac{b}{\Delta T}}$, where µ and *b* are kinetic constants. At the low undercooling ΔT < ΔT*, the *R* is small, and the crystallization process occurs in a step‐growth mode. During the transition regime, ΔT* #x0003C; ΔT < πΔT*, the crystallization is “mock‐lateral” growth. When the ΔT > πΔT*, the *R* will no longer change with increasing ΔT; the crystallization process operates in a continuous mode, featuring a completely rough interface that adversely affects the carrier transport within the device.^[^ ^32^ ^]^ Thus, controlling the crystallization process is achievable through modulation of the undercooling degree. In Stage III (in Figure 1f), the cooling rate decreases, resulting in low undercooling ΔT. A slow cooling rate ensures high‐quality grain growth and recrystallization. As mentioned above, we have delineated two pivotal strategies for regulating perovskite growth and enhancing crystal quality: fast nucleation and slow crystallization, which can be achieved through spontaneous cooling during the preparation process.

**Figure 2:**
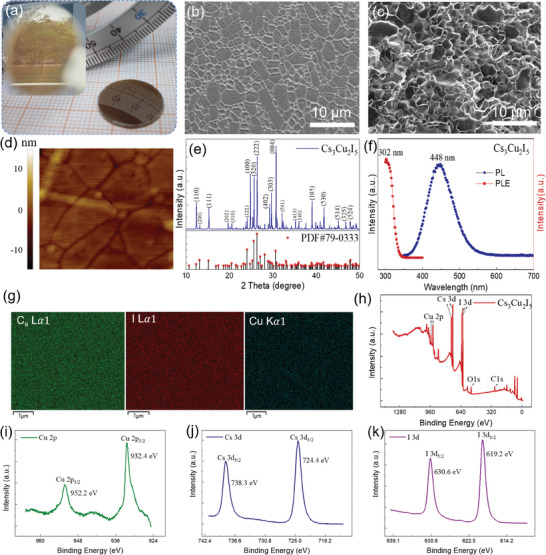
Characterization of the lead‐free perovskite Cs_3_Cu_2_I_5_ wafer. a) Photographs of the Cs_3_Cu_2_I_5_ wafer with a mirror‐like surface. b) Surface and c) cross‐sectional SEM images of the Cs_3_Cu_2_I_5_ wafer. d) AFM image of the Cs_3_Cu_2_I_5_ wafer. The surface roughness R_RMS_ is 4.8 nm. e) XRD diffraction patterns of the Cs_3_Cu_2_I_5_ wafer. f) The PL and PLE spectrum of Cs_3_Cu_2_I_5_ wafer. g) EDS element mapping (Cs, I, and Cu) of the Cs_3_Cu_2_I_5_ wafer, suggesting the uniform distribution of elements. h) XPS survey spectrum of Cs_3_Cu_2_I_5_ wafer. i–k) High‐resolution XPS spectra of (i) Cu 2p, (j) Cs 3d, and (k) I 3d of Cs_3_Cu_2_I_5_ wafer, respectively.

The as‐prepared perovskite wafers exhibit uniformity and integrity at the macroscopic level, with excellent mechanical strength and high surface mirror reflectivity, as shown in **Figure** **2**a. The inset of Figure 2a shows the mirror‐reflection effects of the Cs_3_Cu_2_I_5_ wafer. The objects (ruler, tree, and building) can be clearly displayed by mirror reflection. For the Cs_3_Cu_2_I_5_, the white precursor powders transform into uniform brown semi‐transparent glass wafers after hot pressing, indicating significant grain growth and recrystallization during the hot‐pressing process.

The detailed micromorphology of the Cs_3_Cu_2_I_5_ wafers surface is further investigated by the scanning electron microscope (SEM), as shown in Figure 2b. The perovskite wafers exhibit a uniform and compact surface. The corresponding cross‐sectional SEM (Figure 2c) also shows that the wafers are dense devoid of cavities and constituted by well‐defined large grains, both of which contribute to diminishing charge carrier entrapment at grain boundaries and improving the charge carrier transport.^[^ ^33^ ^]^ To study the surface roughness of the Cs_3_Cu_2_I_5_ wafer, atomic force microscopy (AFM) is performed (Figure 2d), which shows the root mean square average (R_RMS_) of the Cs_3_Cu_2_I_5_ wafer is as low as 4.8 nm, further indicating the uniform surface of the perovskite wafers fabricated by the spontaneous cooling strategy with hot‐pressing technique.

Figure 2e shows the X‐ray diffraction (XRD) profiles for the Cs_3_Cu_2_I_5_ wafer, which exhibits well‐oriented characteristic peaks with narrow full width at half maxima (FWHM) (in Figure S5, Supporting Information), which can be comparable to that of some Cs_3_Cu_2_I_5_ single crystals.^[^ ^34^ ^]^ The characteristic peaks are indexed well by standard data card (PDF#79‐0333), and no additional phase can be identified. Nevertheless, our observations reveal a slight right shift in the diffraction peak of the Cs_3_Cu_2_I_5_ wafer relative to the standard PDF#79‐0333, suggesting a subtle lattice compression. This phenomenon may be due to the high pressure which induces a more compact arrangement of the atoms. As shown in Figure S4 (Supporting Information), compared with microcrystal precursor powder and the standard diffraction pattern, the XRD profiles of the wafer show a difference from that of the powder counterpart in diffraction intensity. The intensity of diffraction peaks increases significantly after the hot‐pressing method, which is related to the improved crystallinity. Additionally, the wafer shows a strong diffraction peak of the (004) plane, while for the powder the strongest diffraction peak is located at the (400) plane, indicating the preferred growth of crystals has undergone changes in the crystal plane during the hot‐pressing process. The cell occupies a minimum volume when all (00*k*) planes are parallel to the horizontal level. Consequently, this observation could be attributed to an increased alignment of (400) planes parallel to the substrate, aiming to reduce the volume occupied under pressure. This phenomenon also suggests that the sintering process promotes a preferential orientation and facilitates recrystallization.^[^ ^35^ ^]^

Figure 2f shows the photoluminescence (PL) and photoluminescence excitation (PLE) spectrum of the Cs_3_Cu_2_I_5_ wafer. It presents a strong PLE peak at 302 nm combined with a broad PL emission peak located at 448 nm, which is consistent with previous reports.^[^ ^36^ ^]^ To further determine the uniformity of chemical elements in the Cs_3_Cu_2_I_5_ wafer, energy‐dispersive spectroscopy (EDS) mapping conducted on an SEM system was performed. As shown in Figure 2g, EDS element mappings reveal a homogenous distribution of elements Cs, I, and Cu. Quantitative analysis by EDS reveals a Cs:I:Cu atomic ratio of 3:5:2, indicating the formation of Cs_3_Cu_2_I_5_ with good stoichiometry.

X‐ray photoelectron spectroscopy (XPS) analysis was carried out to describe the valence state and surface composition of the Cs_3_Cu_2_I_5_ wafer. Figure 2h–k reveals the survey spectrum and the high‐resolution spectra of Cs 3d, Cu 2p, and I 3d components, which are conducted to validate the chemical states of matrix ions, respectively. Two peaks located at 932.4 and 952.2 eV are the characteristic peaks of Cu^+^ 2p_3/2_ and Cu^+^ 2p_1/2_ (Figure 2i), respectively. The binding energies of Cu^+^ 2p_3/2_ and Cu^+^ 2p_1/2_ are consistent with Cu─I bond and demonstrate the presence of Cu^+^ in the host. The absence of a satellite peak precludes the Cu^2+^ oxidation state. In Figure 2j,k, the binding energies of the 3d orbital correspond to the Cs and I +1 and ‐1 states, respectively.^[^ ^37^ ^]^

**Figure 3:**
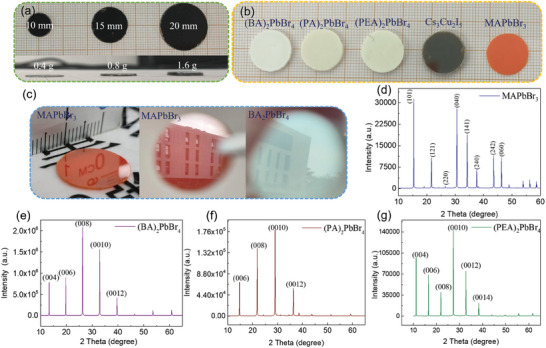
Photographs and XRD diffractograms of organic‐inorganic perovskite MAPbBr_3_ and 2D perovskite BA_2_PbBr_4_, PA_2_PbBr_4_, and PEA_2_PbBr_4_ wafers. a) Photographs of perovskite wafers with diameters of 10, 15, and 20 mm and different thicknesses (0.489, 0.967, and 1.443 mm). b) Optical images of 5 types of perovskite wafers, including BA_2_PbBr_4_, PA_2_PbBr_4_, PEA_2_PbBr_4_, Cs_3_Cu_2_I_5_, and MAPbBr_3_. c) Photographs of MAPbBr_3_ and BA_2_PbBr_4_ wafers with mirror‐like surfaces. d–g) XRD diffractograms of MAPbBr_3_, BA_2_PbBr_4_, PA_2_PbBr_4_, and PEA_2_PbBr_4_, respectively.

The size of the wafer with diameters of 10, 15, and 20 mm can be controlled by changing the mold (**Figure** **3**a), and we think as long as the diameter of the grinding tool is large enough, any large‐size wafers can be prepared. Besides, the thickness of the wafer can also be tailored by controlling the amounts of raw materials. As demonstrated in Figure 3a, the thicknesses of the Cs_3_Cu_2_I_5_ wafer with precursor powder masses of 0.4, 0.8, and 1.2 g are 0.489, 0.967, and 1.443 mm, respectively.

Moreover, spontaneous cooling with a hot‐pressing technique to produce perovskite wafers is not limited to the Cs_3_Cu_2_I_5_ wafer reported above. It is universal for fabricating various perovskites, including organic–inorganic perovskite, 2D perovskite, lead‐free perovskite, etc. Figure 3b shows the photographs of perovskite wafers including BA_2_PbBr_4_, PA_2_PbBr_4_, PEA_2_PbBr_4_, and MAPbBr_3_. The as‐prepared perovskite wafers also exhibit good uniformity with high surface mirror reflection, as shown in Figure 3b. Figure 3c shows the mirror reflection effects of perovskite wafers, which indicates the surface specular reflectivity of these wafers is not inferior to that of Cs_3_Cu_2_I_5_ wafers. Surface and cross‐section SEM images of BA_2_PbBr_4_, PA_2_PbBr_4_, PEA_2_PbBr_4_, and MAPbBr_3_ wafers are shown in Figures S1 and S2 (Supporting Information), respectively. The surface and cross‐section micro‐morphologies of these wafers demonstrate that the prepared wafers have characteristics of large grains and no pinholes.

To characterize the purity and high crystallinity of wafers, XRD diffractograms of MAPbBr_3_, BA_2_PbBr_4_, PA_2_PbBr_4_, and PEA_2_PbBr_4_ are shown in Figure 3d–g, respectively. In Figure 3d, the XRD patterns of the as‐prepared MAPbBr_3_ wafer reveal that the crystalline is consistent with a cubic structure (space group Pm $\bar{3}$ m) as reported previously.^[^ ^38^ ^]^ Figure 3e–g presents the XRD patterns of BA_2_PbBr_4_, PA_2_PbBr_4_, and PEA_2_PbBr_4_ wafers, respectively, where the succession of quasi‐equally spaced (002) diffraction peaks substantiates the 2D orthorhombic crystal structure, which match well with the calculated XRD patterns.^[^ ^39^, ^40^, ^41^ ^]^

According to previous mechanism analysis, temperature and pressure are crucial for preparation of the high‐quality perovskite wafers. We can use AFM results to simply evaluate the quality of wafers. For the Cs_3_Cu_2_I_5_ wafer, with increasing temperature at 50 MPa, we can observe a reduction of surface roughness R_RMS_ from 17.3 nm at room temperature to 4.8 nm at 150 °C (see Figure 2d; Figure S3a, Supporting Information). It is also noticeable that the grain boundaries appear more distinct, and no primary powder can be observed anymore. In contrast, a lower surface quality is observed when the perovskite wafer is pressed at 150 °C and 10 MPa (in Figure S3b, Supporting Information, R_RMS_ = 238.8 nm). At higher pressure, the surface roughness (in Figure S3c, Supporting Information, R_RMS_ = 5.4 nm, at 100 MPa) shows the same level as at medium pressure (R_RMS_ = 4.8 nm, at 50 MPa), without significant improvement. Due to grain growth, reduced pinholes, and decreased surface roughness during hot pressing, we can achieve high‐quality perovskite wafers during pressing at 50 MPa and 150 °C. This also facilitated the formation of clear grain boundaries with good grain boundary interconnection. Therefore, it is deduced that a sufficiently high combination of pressure and temperature is essential for achieving high‐purity, high‐density, pinhole‐free perovskite wafers with good grain‐boundary interconnection.

Furthermore, during the cooling stage, to compare gradient cooling with spontaneous cooling, we set the rate of temperature gradient reduction to ≈0.5 °C min^−1^ and keep all other conditions unchanged. The surface roughness R_RMS_ of the wafer by gradient cooling is 8.1 nm (Figure S3d, Supporting Information), which is higher than that of spontaneous cooling (R_RMS_ = 4.8 nm), indicating that spontaneous cooling is better for enhancing the quality of perovskite wafers. The reason we think is that spontaneous cooling facilitates a more gradual cooling process via a thick‐shelled heating mold. In the traditional annealing process of perovskites, the gradient thermal annealing may be good for achieving higher‐quality crystallization due to the lack of insulated thick shells. However, the existence of the insulation effect of the mold will be more beneficial for the spontaneous cooling strategy to realize high‐quality perovskite wafers. In addition, spontaneous cooling is very simple and low cost, which does not require complex temperature control equipment, reduces energy consumption, and can be more easily applied widely.

**Figure 4:**
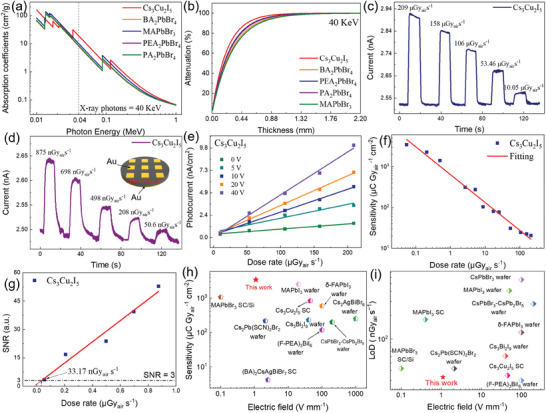
X‐ray detection properties of the wafer detectors. a) Absorption coefficients of typical X‐ray detection materials as a function of photon energy. b) Thickness‐dependent X‐ray attenuation efficiency of typical X‐ray detection materials to 40 KeV X‐ray photons. c,d) The on/off X‐ray photocurrent response biased at 10 V for Cs_3_Cu_2_I_5_ wafer‐based X‐ray detector under different dose rates range from (c) 10.05 to 209 µGy_air_ s^−1^, and (d) 50.6 to 875 nGy_air_ s^−1^, respectively. e) Photocurrent of the Cs_3_Cu_2_I_5_ detector under different dose rates with a bias of 0, 5, 10, 20, and 40 V, respectively. f) Sensitivity of Cs_3_Cu_2_I_5_ X‐ray detectors under different dose rates. g) Dose rate‐dependent SNR of the Cs_3_Cu_2_I_5_ X‐ray detector biased at 10 V. h) Sensitivity and i) LoD comparison of the as‐fabricated device with previously reported X‐ray detectors based on perovskite wafer and single crystal.

The X‐ray detection performances of Cs_3_Cu_2_I_5_, MAPbBr_3_, BA_2_PbBr_4_, PEA_2_PbBr_4_, and PA_2_PbBr_4_ wafers are systematically investigated under vacuum conditions (<1 Pa) to eliminate the impact of air ionization on device performance.^[^ ^42^ ^]^ The devices are illuminated by an X‐ray source with the largest X‐ray energy is 40 KeV. The dose rate has been calibrated by a Radcal dosimeter. **Figure** **4**a,b show the absorption coefficients as a function of X‐ray photon energy and the attenuation efficiency of 40 KeV X‐ray photons as a function of different thicknesses of typical X‐ray detector materials, respectively. The absorption coefficients of perovskites are obtained from the (National Institute of Standards and Technology, NIST) database, and the absorption coefficient of Cs_3_Cu_2_I_5_ is higher than that of MAPbBr_3_, BA_2_PbBr_4_, PEA_2_PbBr_4_, and PA_2_PbBr_4_ with the X‐ray energy of 40 KeV (Figure 4a). In general, the attenuation capacity of iodine‐containing perovskites is stronger than that of brominated perovskites. Therefore, a smaller thickness is enough for the Cs_3_Cu_2_I_5_ wafer to attenuate most X‐rays compared to brominated perovskites. The high absorption coefficient supports that a 0.8 mm thickness of Cs_3_Cu_2_I_5_ wafer can attenuate 97% of X‐ray photons (Figure 4b). Therefore, the 0.8 mm thick wafer is selected and assembled into a vertical structure (Au/Cs_3_Cu_2_I_5_/Au) for further testing, as illustrated in the inset of Figure 4d.

The on/off photocurrent responses biased at 10 V for Cs_3_Cu_2_I_5_ wafer‐based X‐ray detectors are present in Figure 4c,d with the dose rates from 10.05 to 209 µGy_air_ s^−1^ and 50.6 to 875 nGy_air_ s^−1^, respectively. It can be concluded that the Cs_3_Cu_2_I_5_ wafer‐based detector has a steady current signal, good reproducibility, and a stable baseline. The photo and dark current of the Cs_3_Cu_2_I_5_ wafer detector exhibits clear differentiation at an extremely low dose rate of 50.6 nGy_air_ s^−1^ (Figure 4d). Meanwhile, BA_2_PbBr_4_, PEA_2_PbBr_4_, and PA_2_PbBr_4_ wafers exhibit the same characteristics, as shown in Figures S7 and S8 (Supporting Information). In contrast, the dark current keeps increasing for the MAPbBr_3_ wafer with the dose rates from 2.67 to 9.84 µGy_air_ s^−1^, and thus the signal value becomes inaccurate (see Figure S7d, Supporting Information). It indicates that low‐dimensional perovskites exhibit superior stability, which can effectively suppress ion migration within the wafer.

In Figure 4e, the photocurrent exhibits a linear correlation with the X‐ray dose rate under different biases. All wafer detectors exhibit the same characteristics (in Figure S9, Supporting Information). There is a linear relationship between photocurrent and X‐ray dose rate, and the photocurrent increases with the increase of the bias, owing to the better charge collection under higher bias. The sensitivity (*S*) of the Cs_3_Cu_2_I_5_ wafer detector can be calculated according to Equation:^[^ ^43^ ^]^

(3) $$S=\frac{I_{signal}}{DA}=\frac{\bar{I_{photo}}-\bar{I_{dark}}}{DA}$$

where *I_signal_* is the effective device signal current, $\bar{I_{photo}}$ is the average device current under X‐ray irradiation, $\bar{I_{dark}}$ represents the average dark current, *D* is the dose rate, *A* is the effective area of the device. As illustrated in Figure 4f, there is a monotonic decrease in sensitivity as the dose rate increases, attributable to the filling of deep defect states^[^ ^44^ ^]^ and exciton–exciton annihilation^[^ ^45^ ^]^ as previously reported.^[^ ^46^ ^]^ The predominant mechanism for sensitivity enhancement at low dose rates is photoconductive gain, which is facilitated by defect‐related traps. These traps capture charge carriers, thereby extending their recombination lifetimes, which supports the accumulation of carriers and leads to effective charge multiplication. However, the mere existence of a significant distribution of traps does not guarantee photoconductive gain; two additional conditions are imperative: 1) a high internal electric field must be present to ensure efficient de‐trapping of the carriers, and 2) the photon flux (φ) must be sufficiently low to prevent the complete filling of the trap levels. In the experiments conducted in this work, the requisite high internal electric field condition was consistently satisfied under the applied biases. Conversely, at high dose rates, trap levels tend to fill progressively, leading to increasing dominance of band‐to‐band recombination over trap‐assisted recombination, thereby progressively diminishing the photoconductive gain.^[^ ^46^ ^]^ Therefore, the sensitivity can reach as large as 3343.6 µC Gy_air_^−1^ cm^−2^ under a low dose rate of 50.6 nGy_air_ s^−1^ at 10 V bias, which is almost 160 times higher than that for the α‐Se X‐ray detectors (20 µC Gy_air_^−1^ cm^−2^ at 10 V). Moreover, with the increase of bias from 0 to 40 V, there is a progressive enhancement in sensitivity, attributed to the elevated electric field that facilitates superior charge collection efficiency and photoconductive gain (in Figure S10, Supporting Information). Besides, in Figure S11 (Supporting Information), the sensitivity of the BA_2_PbBr_4_, PA_2_PbBr_4_, and PEA_2_PbBr_4_ wafer detectors is 544.83, 827.29 and 3416.41 µC Gy_air_^−1^ cm^−2^, respectively, which are obtained under a low dose rate of 50.6 nGy_air_ s^−1^ at 10 V bias. In addition, the theoretical sensitivity (*S_0_*) without considering the photoconductive gain of the perovskite wafer detectors was calculated. The detailed calculation process is provided in the Supporting Information section. According to the calculation, the theoretical sensitivity *S_0_* (the case without gain) of Cs_3_Cu_2_I_5_, BA_2_PbBr_4_, PA_2_PbBr_4_, and PEA_2_PbBr_4_ wafer detectors is 1.12 ×  10^3^ µC Gy_air_^−1^ cm^−2^, 1.28 ×  10^3^ µC Gy_air_^−1^ cm^−2^, 1.3 ×  10^3^ µC Gy_air_^−1^ cm^−2^, and 1.33 ×  10^3^ µC Gy_air_^−1^ cm^−2^, respectively. The sensitivity of Cs_3_Cu_2_I_5_ and PEA_2_PbBr_4_ wafer X‐ray detector in this work is higher than *S_0_*, which further confirms the high gain of these X‐ray detectors.

Additionally, the limit of detection (LoD) is also an essential parameter for assessing the X‐ray detector performance, which is equivalent to the dose rate that produces a signal three times greater than the noise level, and the low detection limit is advantageous for minimizing the radiation exposure experienced by individuals during X‐ray diagnostic procedures. The signal‐to‐noise ratio (SNR) values were calculated using the following equations:^[^ ^35^ ^]^

(4) $$SNR=\frac{I_{signal}}{I_{noise}}$$

(5) $$I_{signal}=\bar{I_{photo}}\;-\bar{I_{dark}}$$

(6) $$I_{noise}=\sqrt{\frac{\sum_{i=1}^{N}{\left(I_{i}-\bar{I_{photo}}\right)}^{2}}{N}}\;$$

where *I_noise_* is the effective device noise current, and *N* is the number of parallel experiments at each bias. The LoD of 33.17 nGy_air_ s^−1^ is derived from the linear fitting with an SNR of 3 shown in Figure 4g, indicating its high detection ability at low radiation doses, which is significantly below the threshold for traditional medical imaging applications (5.5 µGy_air_ s^−1^). The LoD of BA_2_PbBr_4_, PA_2_PbBr_4_, and PEA_2_PbBr_4_ wafer detectors is 523.3, 60, and 27 nGy_air_ s^−1^, respectively, as shown in Figure S11 (Supporting Information). Figure 4h,i summarize the sensitivity and LoD comparison of the Cs_3_Cu_2_I_5_ wafer detector with other perovskite‐based polycrystalline wafer and single crystal (SC) X‐ray detectors reported previously.^[^ ^35^, ^47^, ^48^, ^49^, ^50^, ^51^, ^52^, ^53^, ^54^, ^55^, ^56^, ^57^, ^58^ ^]^ It can be seen that the sensitivity of Cs_3_Cu_2_I_5_ wafer X‐ray detector exceeds most of the reported X‐ray sensitivity of detectors based on Pb‐free perovskite polycrystalline wafer and SC, and the LoD of this work is among the lowest levels of in all kinds of perovskite X‐ray detectors, together confirming the high‐quality of the Cs_3_Cu_2_I_5_ wafer. The high sensitivity and low LoD demonstrate the potential application prospects of our perovskite wafers in X‐ray detection.

**Figure 5:**
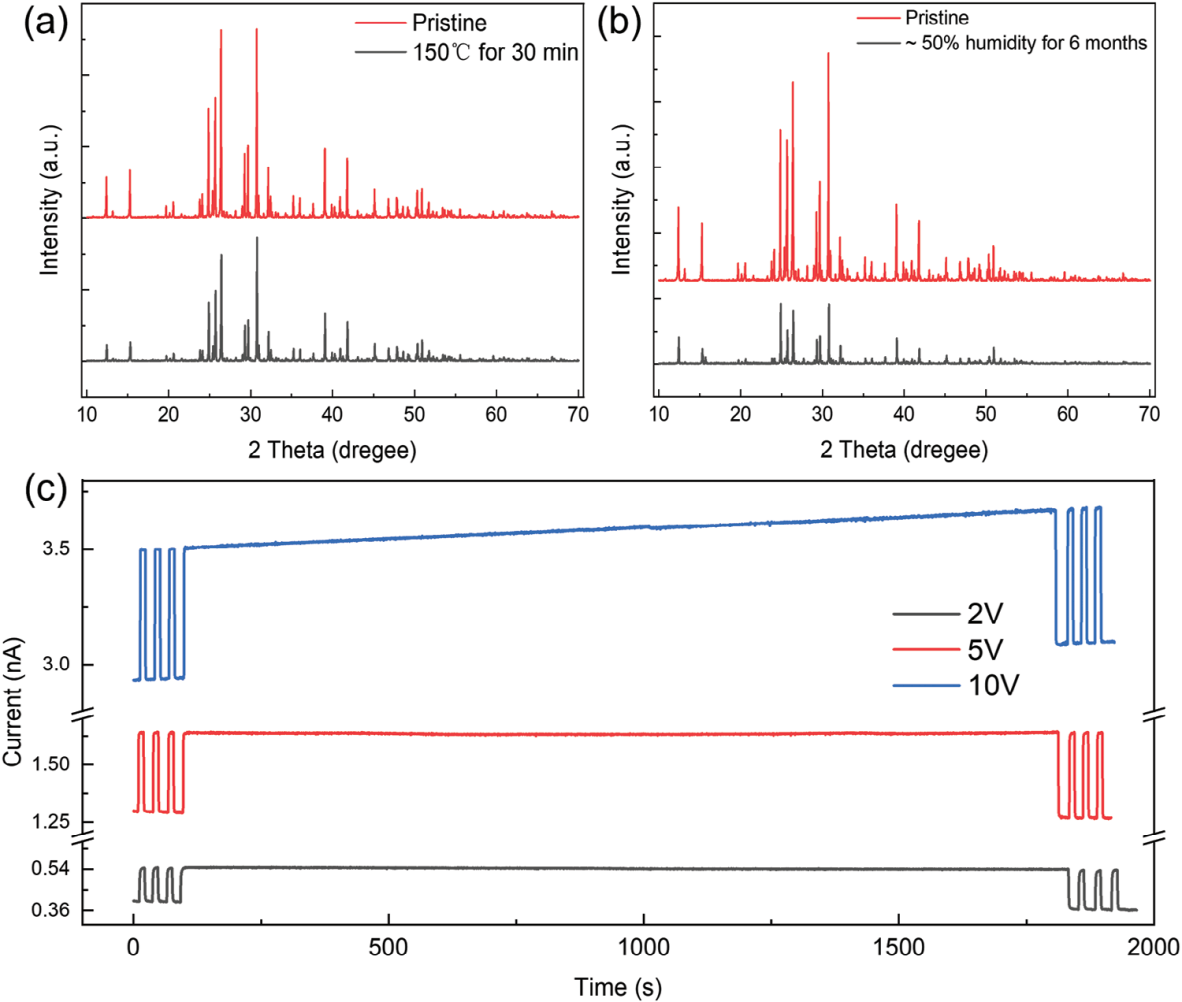
The stability of the Cs_3_Cu_2_I_5_ wafers. a) XRD patterns of Cs_3_Cu_2_I_5_ wafer before and after storage under ambient conditions with ≈50% humidity for 6 months, b) after annealing at 150 °C for 30 min. c) Long‐term irradiation stability tests of Cs_3_Cu_2_I_5_ wafer detector with dose rates of 209 µGy_air_ s^−1^ at a bias of 2, 5, and 10 V, respectively.

The ambient and thermal stability of the Cs_3_Cu_2_I_5_ wafer is evaluated, as shown in **Figure** **5**. The XRD patterns of the wafers after storage in ambient air with 50% RH for 6 months at room temperature in Figure 5a exhibit no obvious change except a minor reduction in peak intensity, demonstrating good stability in ambient air. In Figure 5b, the Cs_3_Cu_2_I_5_ wafer exhibits good thermal stability in ambient air under 150 °C for 30 min, there are no additional diffraction peaks detected in the XRD pattern, demonstrating no noticeable decomposition is observed. Beyond environmental robustness, sustained operational reliability during exposure to radiation and under‐biased voltage is equally vital for practical applications. The stability of the detectors under varying bias conditions was further evaluated through continuous X‐ray irradiation at a specific dose rate of 209 µGy_air_ s^−1^, as shown in Figure 5c. The photocurrent response of the devices exhibits almost no change up to 1800 s under continuous X‐ray irradiation with the bias of 2 and 5 V, respectively. In contrast, at a bias of 10 V, the dark current of the Cs_3_Cu_2_I_5_ wafer detector shows a 5.7% increase in a test period of ≈1800 s, but the net response current shows little difference throughout the whole test period. The dark current drift under high electric fields is closely related to the ion migration phenomenon in perovskite material. The results highlight the remarkable stability of the Cs_3_Cu_2_I_5_ wafer at challenging ambient conditions without any encapsulation, and the stable response to X‐ray under the 5 V bias, indicating that the long‐term operational stability at low‐bias of the Cs_3_Cu_2_I_5_ wafer.

The X‐ray imaging performance of Cs_3_Cu_2_I_5_ wafer detectors is also investigated by employing a point‐to‐point x‐y scanning imaging system, as illustrated in the schematic diagram of Figure S15 (Supporting Information). The target object is placed between the X‐ray source and the Cs_3_Cu_2_I_5_ wafer detector with an effective electrode area of 9 mm^2^. Throughout the imaging procedure, the X‐ray source and detector remained stationary, and the object was moved via an x‐y 2D position manipulator with a moving step of 1.7 mm. An image of the object is acquired by converting the location‐related transmitted X‐ray photons response current *I (x, y)* into a matrix with grayscales. As shown in Figure S15b (Supporting Information), the L‐shaped wrenches hidden in a plastic box can be clearly imaged under a dose rate of 209 µGy_air_ s^−1^. At a lower dose rate of 10.05 µGy_air_ s^−1^, the shape of the screw can also be clearly distinguished (in Figure S15c, Supporting Information). All these results demonstrate the potential application of Cs_3_Cu_2_I_5_ in the X‐ray imaging field.

## 3 Conclusion

In summary, we introduce a spontaneous cooling strategy with a hot‐pressing technique to fabricate high‐purity, wafer‐scale, pinhole‐free perovskite wafers with mirror‐like reflective surfaces, and reveal the spontaneous cooling mechanism for improving the quality of perovskite wafers. There are two key ways to control perovskite growth and improve the quality of crystals: fast nucleation and slow crystallization, which can be achieved through spontaneous cooling during the preparation process. Additionally, the method can be expanded to various perovskite materials including organic–inorganic perovskite, 2D perovskite, and lead‐free perovskite. These superior perovskite wafers have been effectively used in X‐ray detection. Specifically, under a 10 V bias, our lead‐free Cs_3_Cu_2_I_5_ perovskite wafer X‐ray detector achieves a sensitivity of 3433.6 µC Gy_air_^−1^ cm^−2^ with a detection limit lowered to 33.17 nGy_air_ s^−1^, both of which are the best for Cs_3_Cu_2_I_5_ X‐ray detectors and comparable to other Pb‐based perovskite X‐ray detectors. Furthermore, the Cs_3_Cu_2_I_5_ wafer exhibits remarkable environmental stability and long‐term operational stability without any encapsulation. Our research offers a spontaneous cooling strategy to achieve wafer‐scale, high‐purity, pinhole‐free perovskites with mirror‐like surfaces for X‐ray detection, which paves the way for the integration of perovskite materials into electronic and optoelectronic devices, and makes perovskite wafer a strong competitor in the future X‐ray detection market.

## 4 Experimental Section

### Materials

PABr (Propylamine hydrobromide, ≥99.5%), PEABr (phenethylammonium bromide, ≥99.5%), and BABr (butylammonium bromide, ≥99.5%) were purchased from Xi’ a Polymer Light Technology Co., Ltd. (China). PbBr_2_, CsI (99.9%), and CuI (99.9%) were purchased from Aladdin Reagent Ltd. (China). Diethyl carbonate (≥99%), *N,N*‐dimethylformamide (DMF, analytical reagent), and Isopropyl Alcohol (analytical reagent) were purchased from Shanghai Chemical Reagent Co., Ltd. (China). All reagents were used as received.

### Perovskite Microcrystal Synthesis

The perovskite microcrystals were synthesized by a precipitation reaction. A precursor solution (0.1 m L^−1^) was prepared by dissolving a corresponding molar ratio in DMF at 25 °C and stirred for 6 h. The filtered precursor solution was added to Diethyl carbonate gradually with stirred at 110 °C for 30 min. The microcrystals were washed twice with isopropyl alcohol and then dried in a vacuum oven at 60 °C overnight.

### Preparation of Perovskite Wafer

Perovskite microcrystals were grinded for 5 min to obtain a uniform powder. The powders were then mounted into a hot‐pressing mold, which are sandwiched between two quartz glass spacers. Both sides of the glass are polished stainless‐steel cylinders, which were subjected to a pressure of 100 MPa through a hydraulic press. The mold was equipped with a heating mat. Before applying pressure, the hot‐pressing mold was first heated to 150 °C with an elevation rate of 10 °C min^−1^ and held for 15 min to soften perovskite grains. Then pressure was applied to the perovskite powder using a 10 MPa min^−1^ increase until a certain target pressure (e.g., 100 MPa) was reached. Upon reaching the target pressure for the first time, the setup was kept at the set temperature and pressure for 30 min. In this period, the pressure decreased over time, which made it necessary to regulate it manually to ensure it remained at the targeted pressure level (the pressure fluctuation range does not exceed 10 MPa). The relaxation time of pressure increases with the increase of pressure regulation times. Afterward, the pressure was no longer maintained, and the heating mat was switched off after 45 min. Allow the mold to spontaneously cool under natural conditions. After being cooled to room temperature, the pressure was manually released to allow for demoulding and the perovskite wafer could be taken out with a mirror‐like reflective surface. The size of the wafer could be easily controlled by altering the mold dimensions, and the desired thickness can be achieved by manipulating the quantity of precursor material loaded.

### Device Fabrication, X‐ray Detector Performance Measurement, and X‐ray Imaging

A 0.8 mm‐thick perovskite wafer was utilized for device assembly, where Au/perovskite wafer/Au was stacked vertically. Au electrodes with a thickness of 80 nm were thermally evaporated on both sides of the wafer with set sizes (9 mm^2^). Then, the devices were mounted on an optical cryostat with a 1 Pa low‐pressure chamber to avoid sensitivity overestimation by air ionization and prevent the oxidation of the samples.

An X‐ray tube with a tungsten anode (Newton Scientific. Inc, Mini X2) was used as the source. A Keithley 2450 provided the bias voltage and recorded the response current. The X‐ray source was under a constant 40 KeV voltage. The current was changed from 10 to 200 µA. Al foil (2–16 mm thick) was used as the attenuator between the X‐ray tube and perovskite wafer. The radiation dose rate was assessed utilizing a Radcal ionization chamber (model: 10 × 6‐6), as the dosimetric instrument. During the calibration process, it was essential to ensure that the dosimeter probe was fully covered by X‐rays and positioned 15 cm from the X‐ray tube. Calibration was performed within the ionization chamber (non‐vacuum). The instrument provides a real‐time display of the current dose rate, averaging the dose rate over 60 s to minimize error. The measurement was conducted in darkness and within a vacuum environment to mitigate interference from ambient light and air ionization effects. For acquiring X‐ray imaging, the object was adhered to a x‐y scanning stage (Zolix PSA200‐11‐X), and the object's movement was directed along the x and y axes for precise manipulation.

## Conflict of Interest

The authors declare no conflict of interest.

## Supporting information



Supporting Information

## Data Availability

The data that support the findings of this study are available from the corresponding author upon reasonable request.
