# Supplementary material for: Spontaneous Cooling Enables High‐Quality Perovskite Wafers for High‐Sensitivity X‐Ray Detectors with a Low‐Detection Limit
Source: Adv Sci (Weinh). 2024 Oct 21;11(46):2410303. doi: 10.1002/advs.202410303 (PMC11633536; doi:10.1002/advs.202410303)
Supplement: Supplementary file 1 — Supporting Information [file ADVS-11-2410303-s001.docx]

**Spontaneous Cooling Enables High-Quality Perovskite Wafers for High Sensitivity** **X-Ray Detectors with a Low-Detection Limit**

*Wenyi Wu,^1^ Jianqiang Zhang,^1^ Ciyu Liu,^1^ Jiankai Zhang,^1^ Hoajie Lai**,^1^ Zhongqiang Hu,^2^ and Hai Zhou,^∗, 1^*

*^1^International School of Microelectronics, Dongguan University of Technology, Dongguan 523808, Guangdong, P. R. China*

*^2^School of Electronic Science and Engineering, Xi'an Jiaotong University, Xi'an 710049, Shaanxi, P. R. China*

*^*^Corresponding Author, E-mail addresses: hizhou@dgut.edu.cn (H. Zhou)*


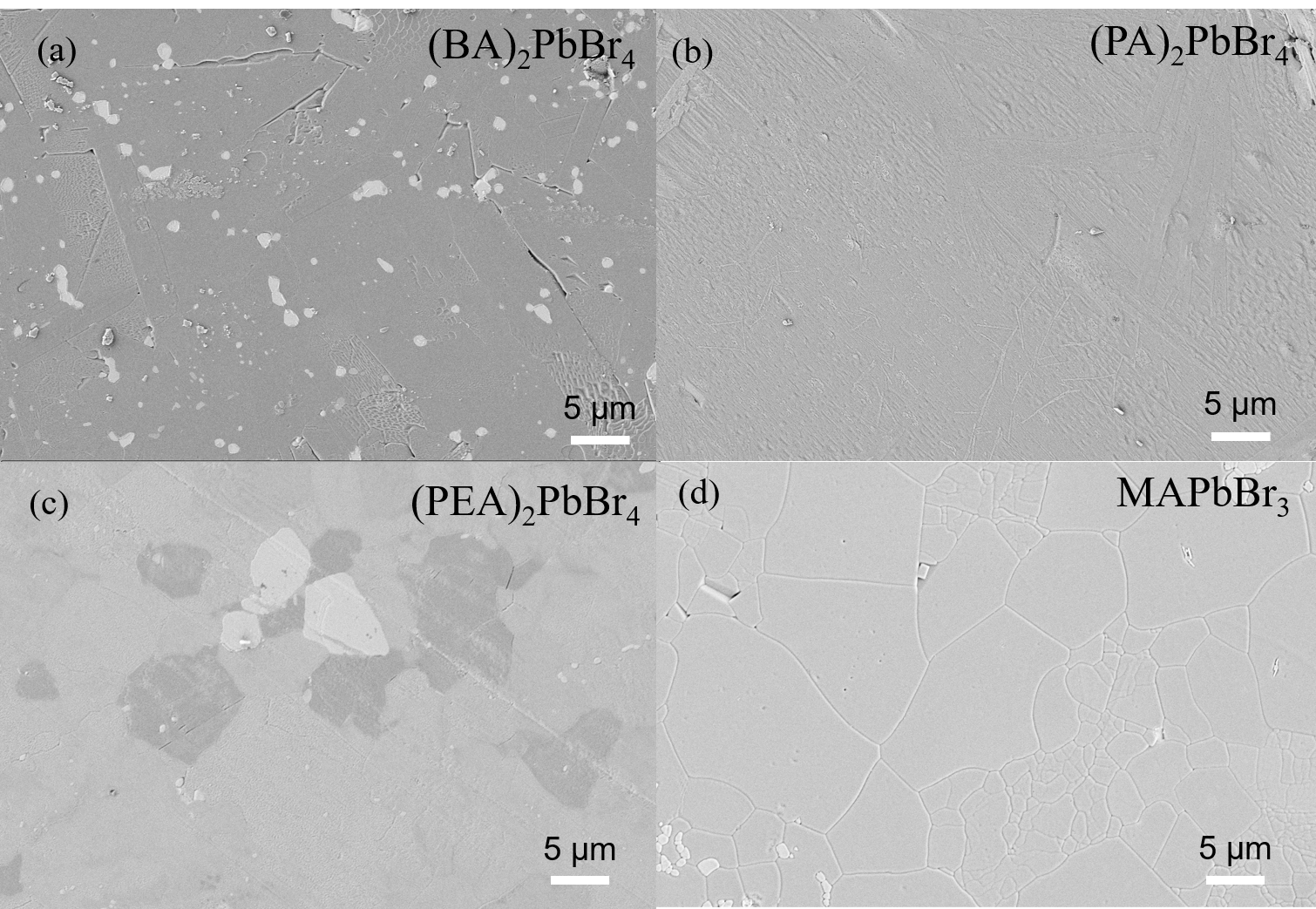


Figure S1 SEM images of surface of BA_2_PbBr_4_, PA_2_PbBr_4_, PEA_2_PbBr_4_, and MAPbBr_3_ wafer, respectively.


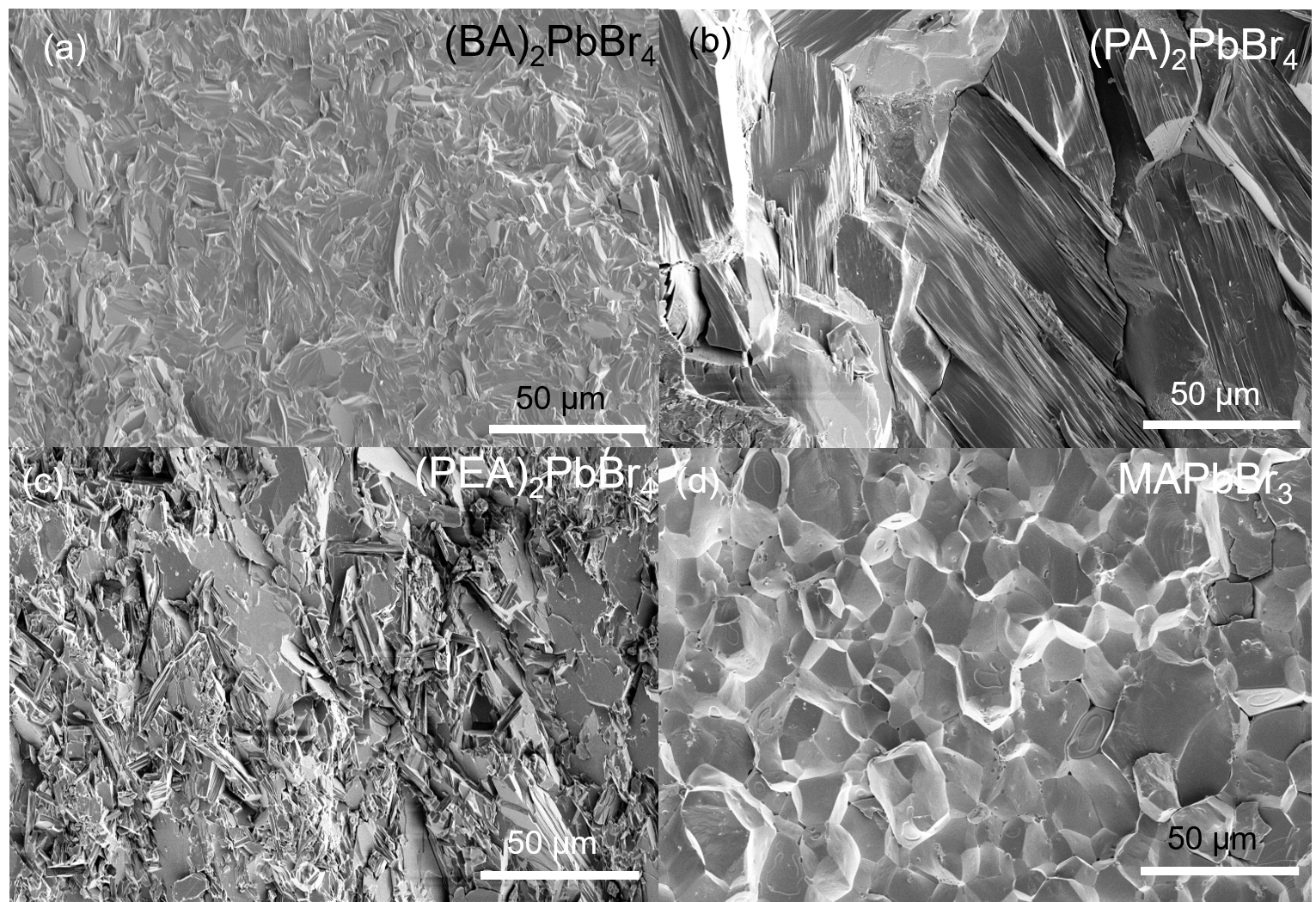


Figure S2 SEM images of the cross-section of BA_2_PbBr_4_, PA_2_PbBr_4_, PEA_2_PbBr_4_, and MAPbBr_3_ wafer, respectively.


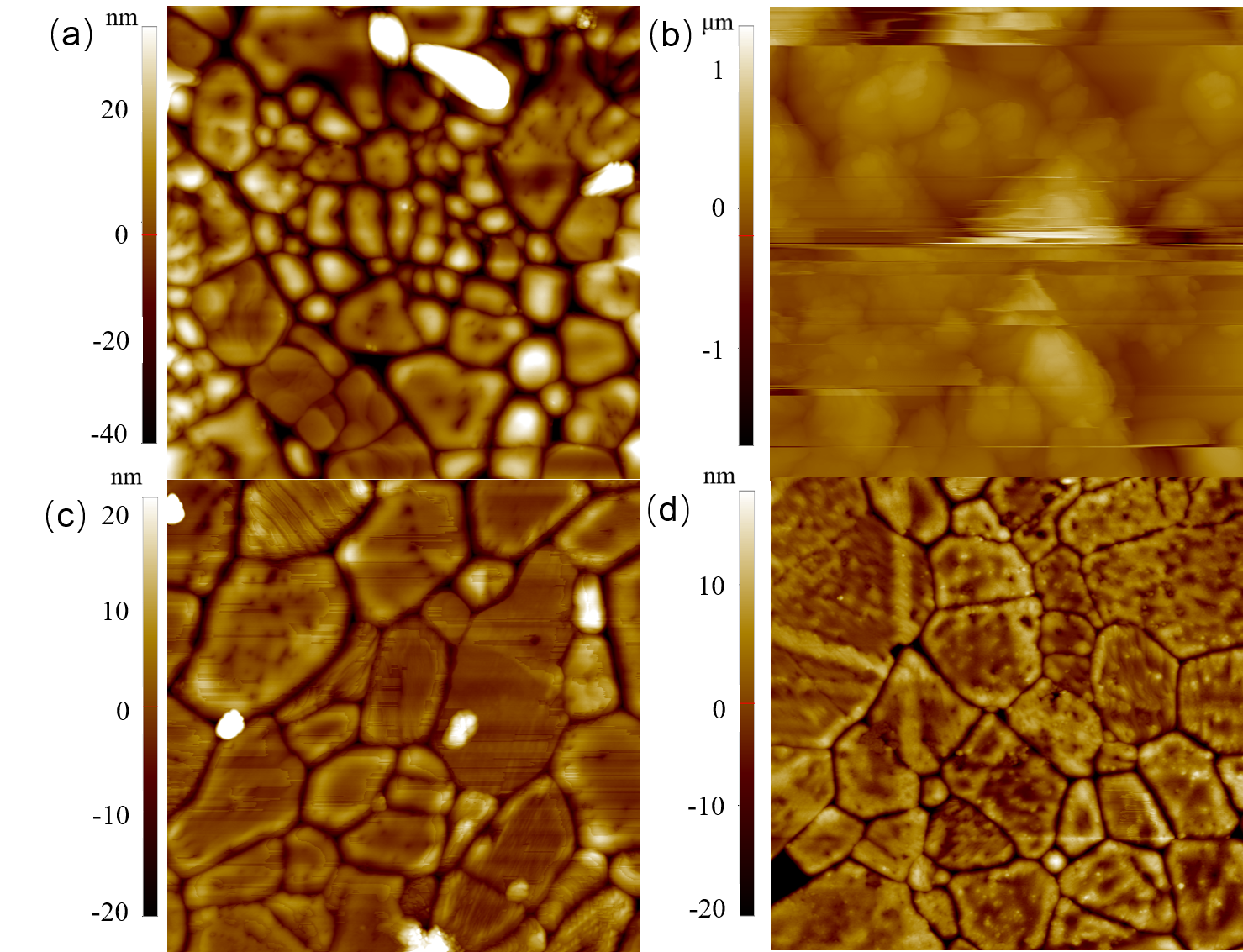


Figure S3 AFM image of Cs_3_Cu_2_I_5_ wafer surface under different preparation conditions. (a) hot-pressing at 50 MPa and room temperature. (b) at 10 MPa and 150 °C (c) at 100 MPa and 150 °C. (d) at 50 MPa and 150 °C, and gradient cooling at a rate of ∼0.5 °C/min. The surface roughness R_RMS_ is 17.3 nm, 238.8 nm, 5.4 nm, and 8.1 nm, respectively.


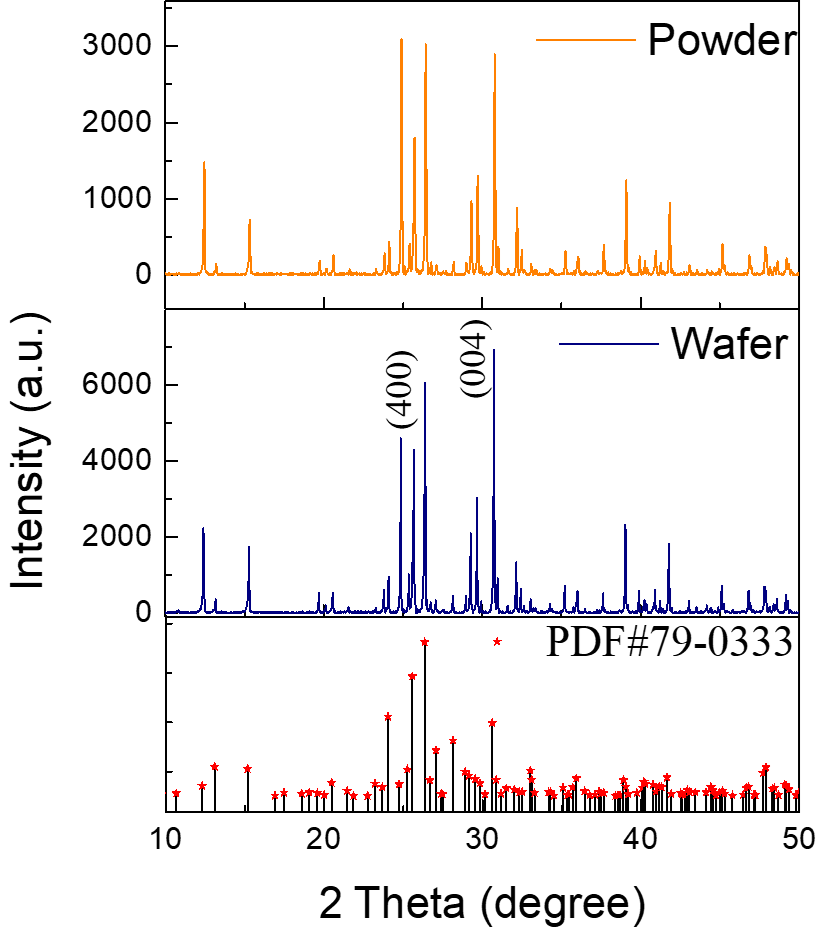


Figure S4 XRD diffractograms of Cs_3_Cu_2_I_5_ wafer and microcrystal precursor powder.


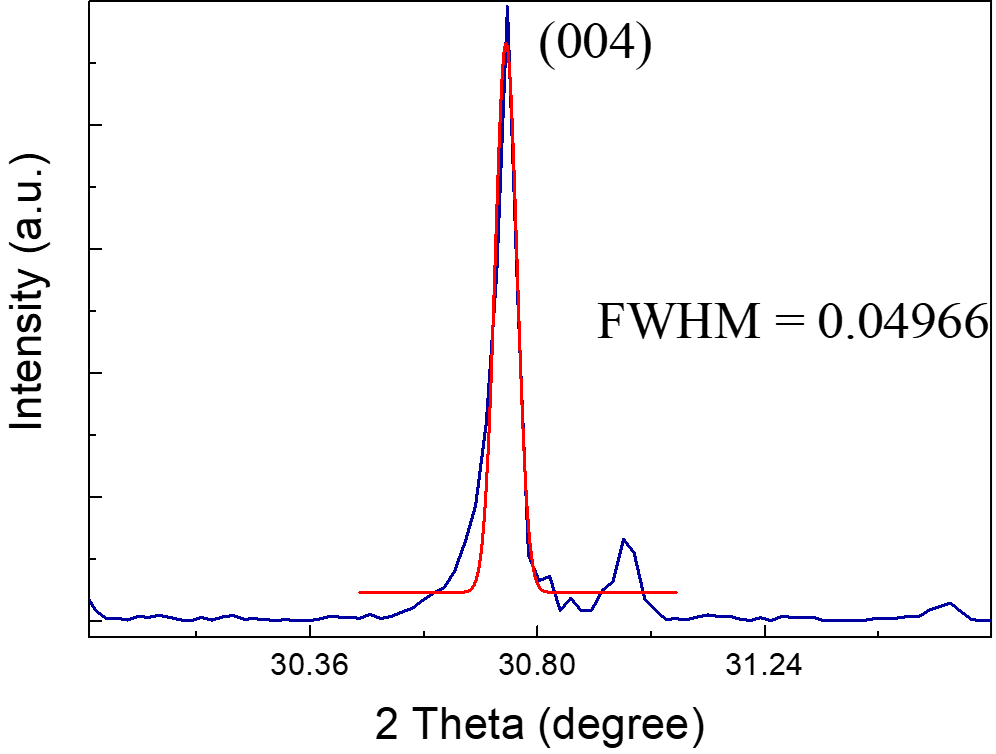


Figure S5 FWHM fitting for XRD pattern of (004) characteristic peak on Cs_3_Cu_2_I_5_ wafer.


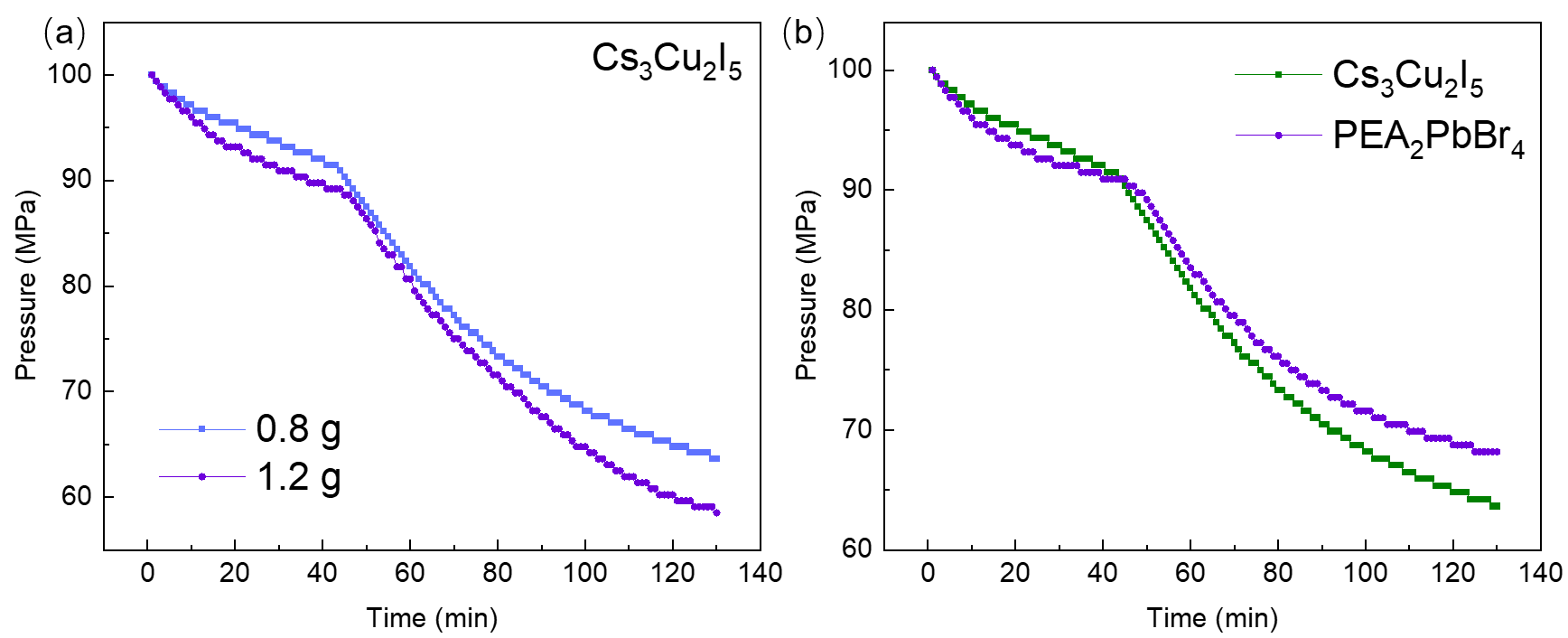


Figure S6 (a) Pressure-time variation curves of Cs_3_Cu_2_I_5_ microcrystalline powders with different masses (0.8 g and 1.2 g) during the hot-pressing process. (b) Pressure-time variation curves for 0.8 g of Cs_3_Cu_2_I_5_ and PEA_2_PbBr_4_ microcrystalline powders during the hot-pressing process.


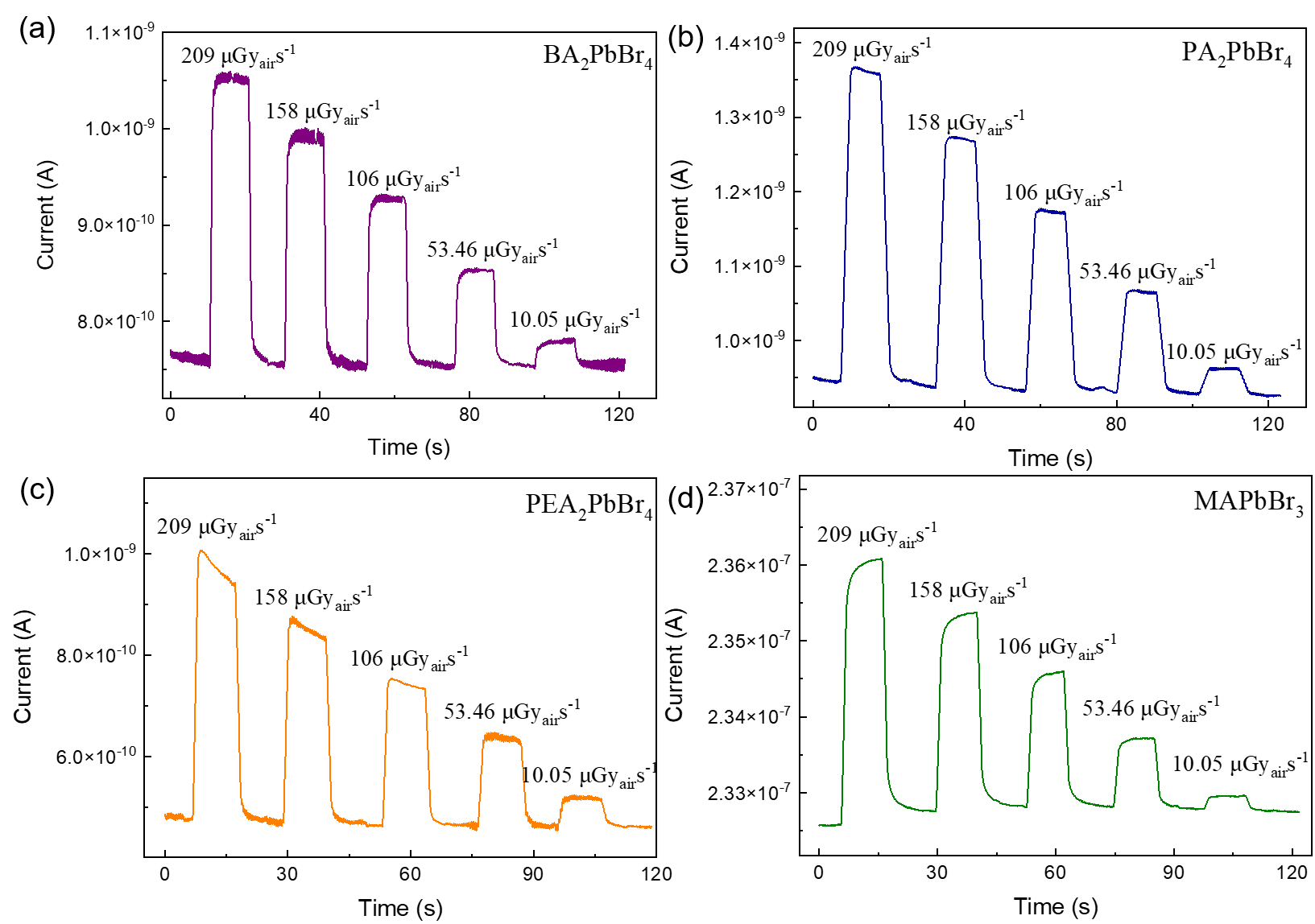


Figure S7 The on/off X-Ray photocurrent response biased at 10 V for (a) BA_2_PbBr_4_, (b) PA_2_PbBr_4_, (c) PEA_2_PbBr_4_, and (d) MAPbBr_3_ wafer-based X-ray detectors under different dose rates range from 10.05 to 209 μGy_air_ s^−1^ under biased at 10 V.


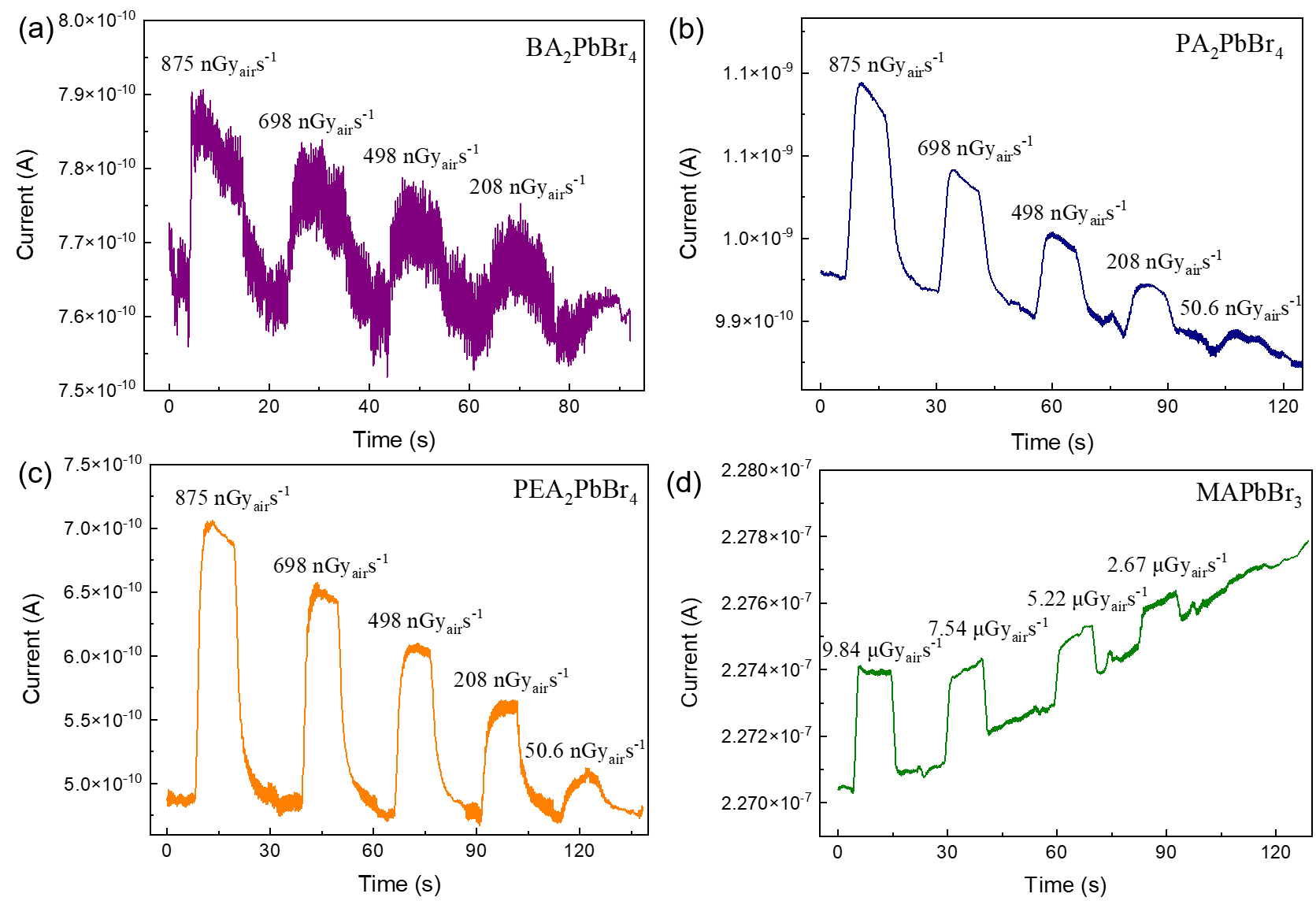


Figure S8 The on/off X-Ray photocurrent response biased at 10 V for (a) BA_2_PbBr_4_, (b) PA_2_PbBr_4_, (c) PEA_2_PbBr_4_, and (d) MAPbBr_3_ wafer-based X-ray detectors under different dose rates range from 50.6 to 875 nGy_air_ s^−1^ under biased at 10 V.


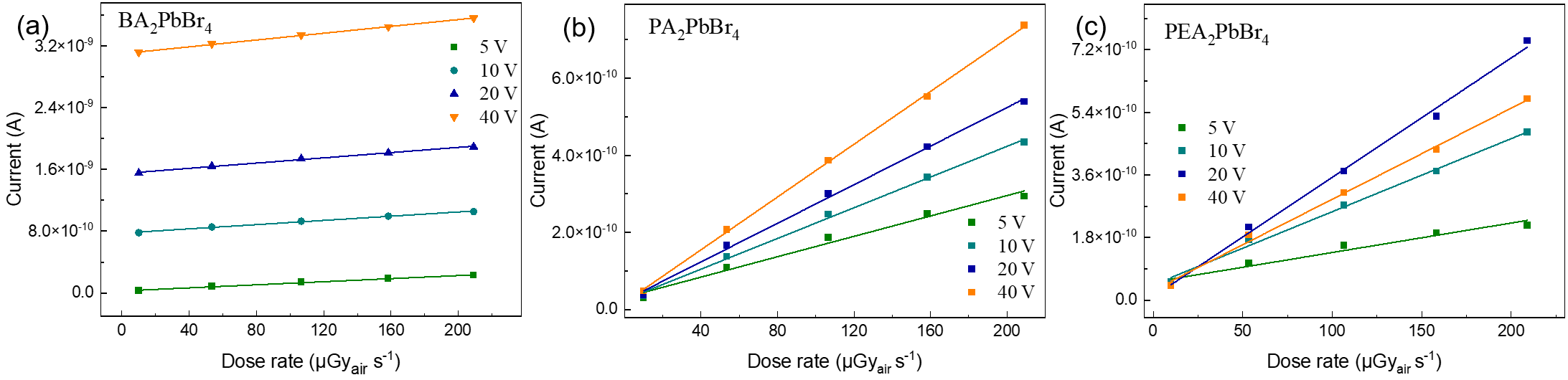


Figure S9 Photocurrent of the (a) BA_2_PbBr_4_, (b) PA_2_PbBr_4_, and (c) PEA_2_PbBr_4_ detector under different dose rates with a bias of 0, 5, 10, 20, and 40 V, respectively.


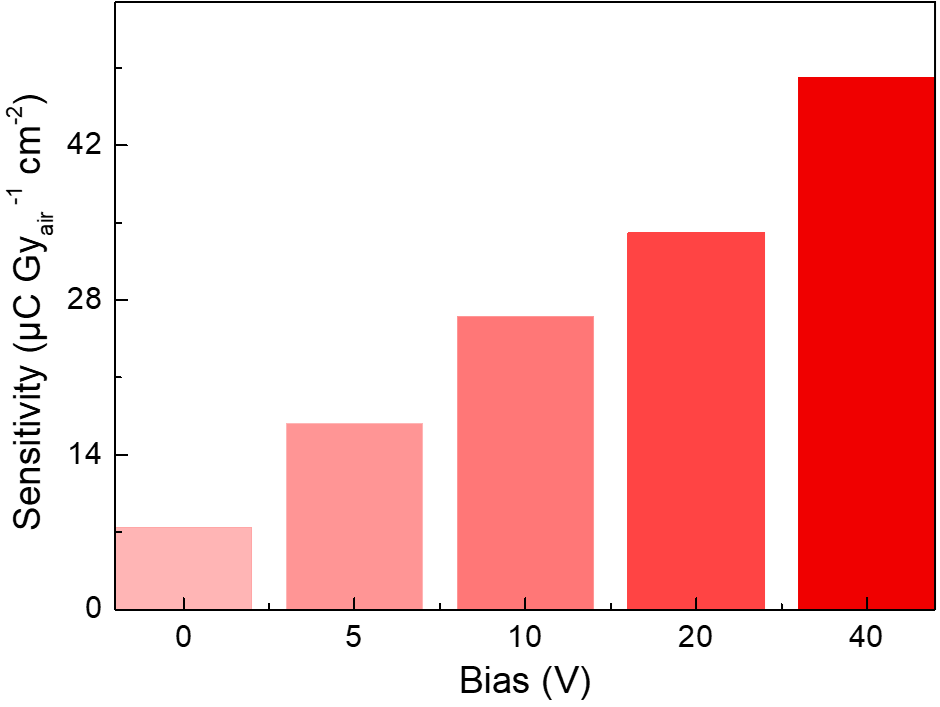


Figure S10 The sensitivity of Cs_3_Cu_2_I_5_ X-ray detector under dose rates of 209 μGy_air_ s^−1^ with a bias of 0, 5, 10, 20, and 40 V.


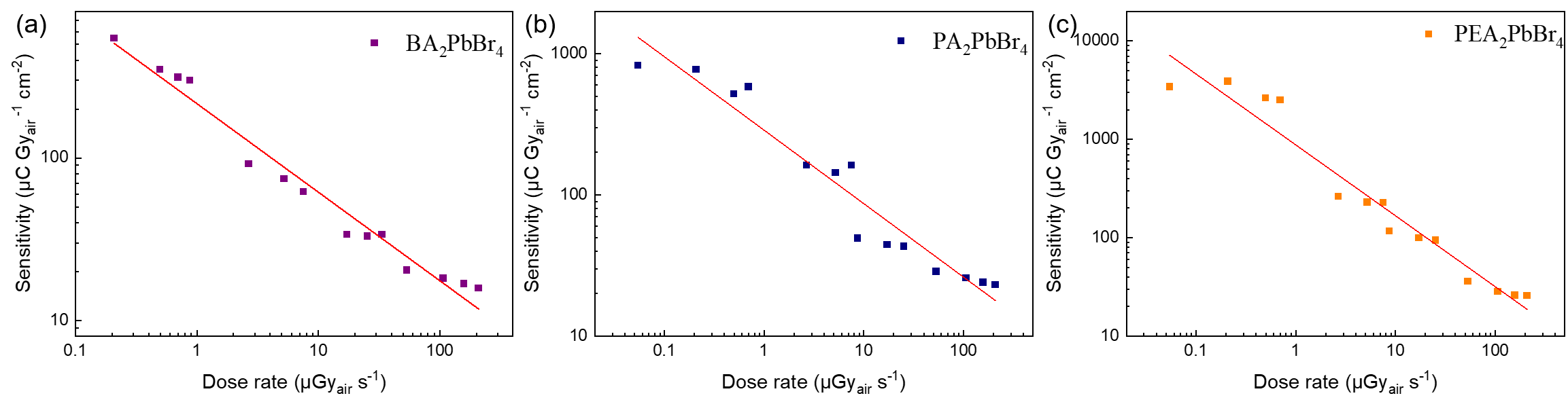


Figure S11 Dose rate-dependent sensitivity of (a) BA_2_PbBr_4_, (b) PA_2_PbBr_4_, and (c) PEA_2_PbBr_4_ X-ray detector under different dose rates with biased at 10 V.


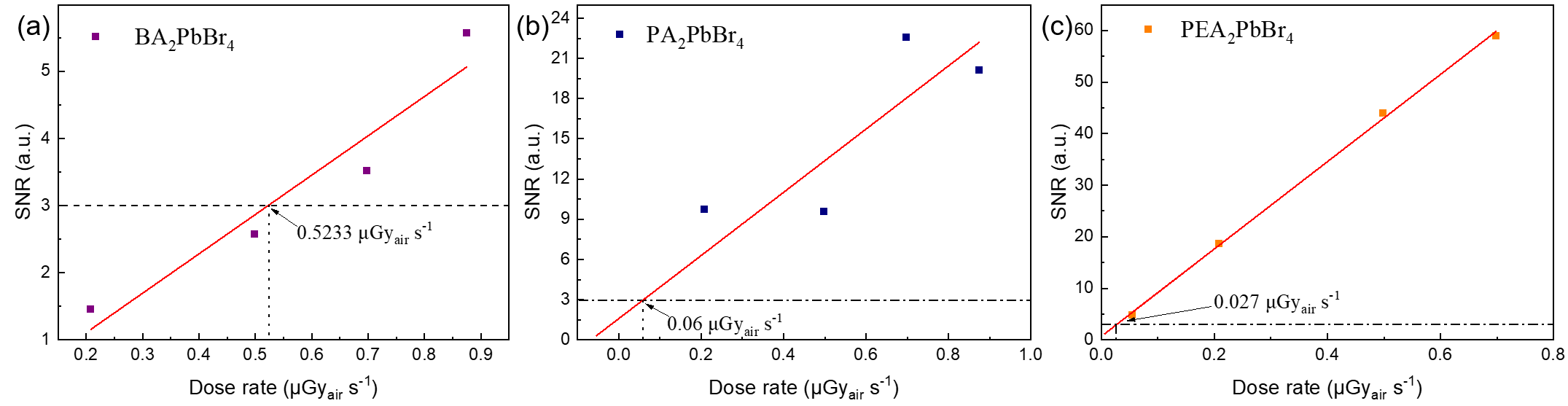


Figure S12 Dose rate-dependent SNR of the (a) BA_2_PbBr_4_, (b) PA_2_PbBr_4_, and (c) PEA_2_PbBr_4_ X-ray detector biased at 10 V.


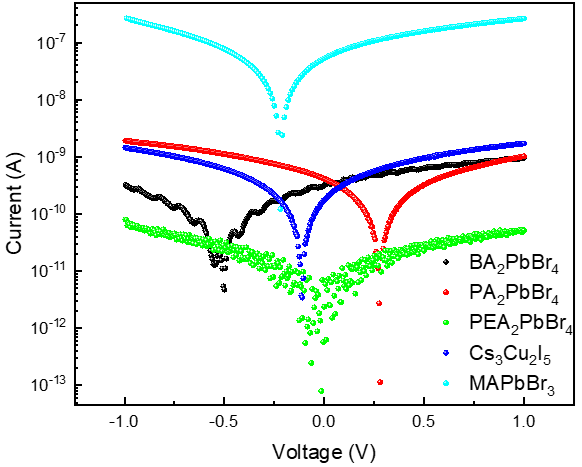


Figure S13 The I-V curves of wafer BA_2_PbBr_4_, PA_2_PbBr_4_, PEA_2_PbBr_4_, Cs_3_Cu_2_I_5_, and MAPbBr_3_ wafer, respectively.


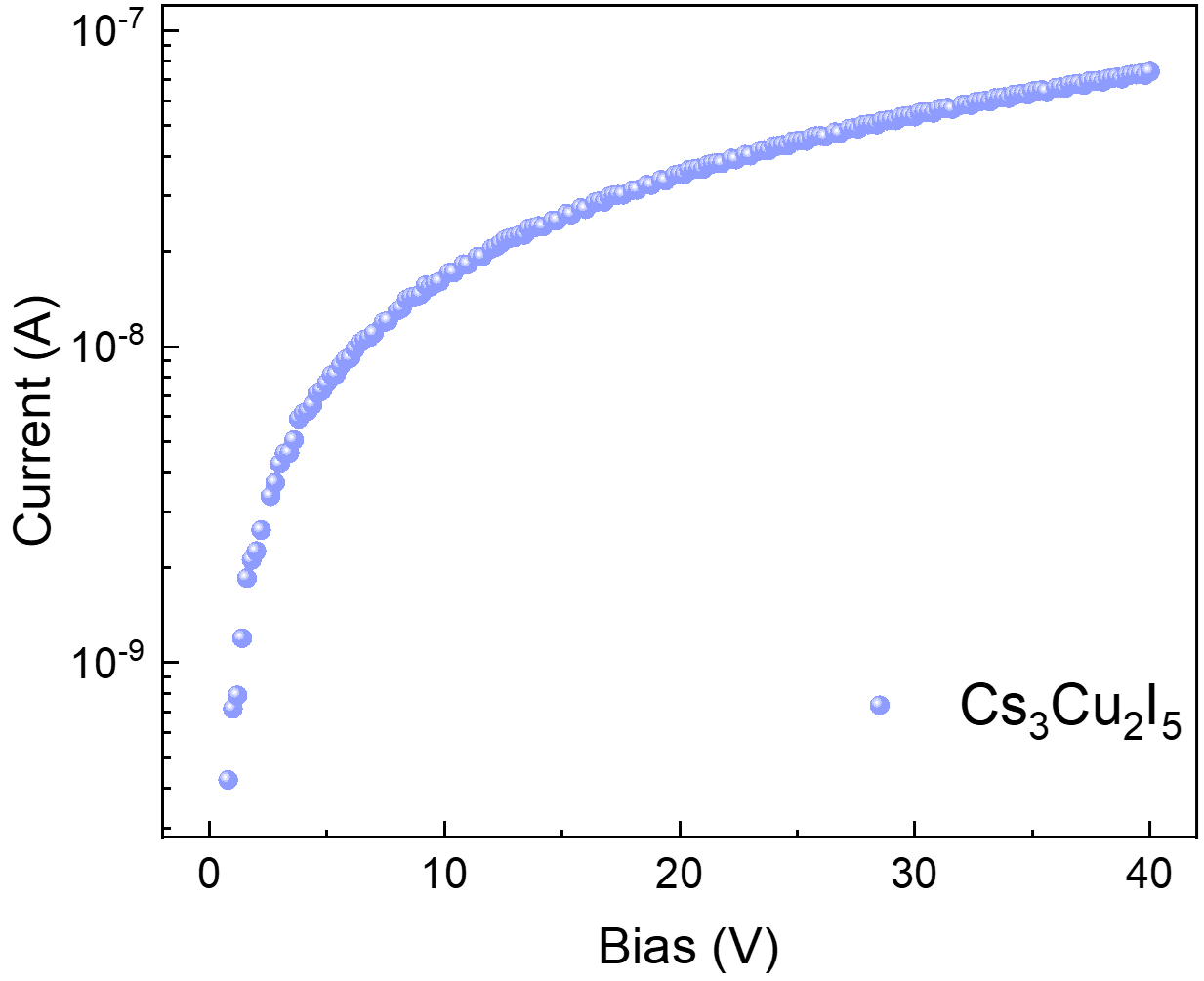


Figure S14 I-V curve of the Cs₃Cu₂I₅ wafer X-ray detector under bias voltages ranging from 0 to 40 V.


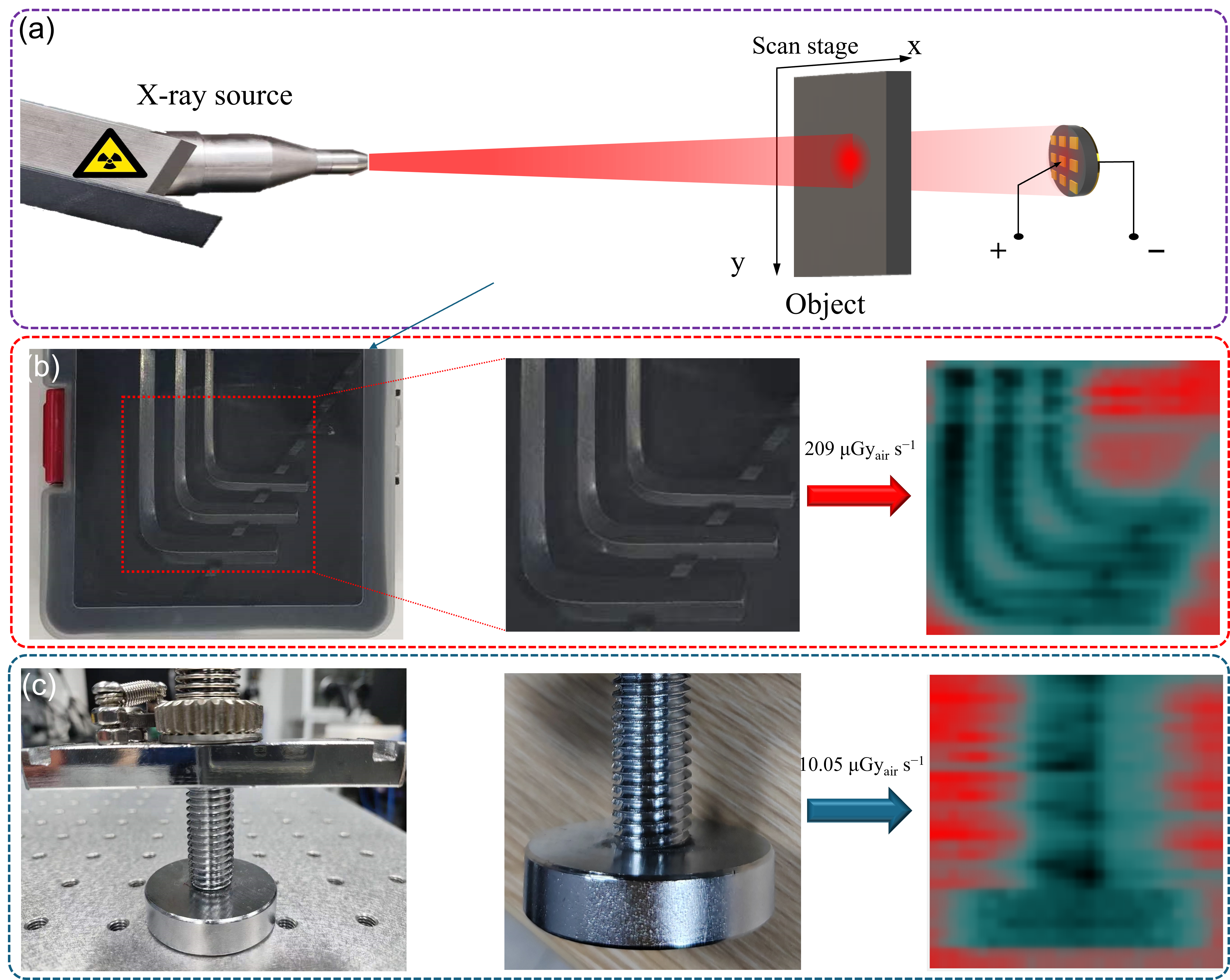


Figure S15 (a) Schematic of the imaging process using the prepared Cs_3_Cu_2_I_5_ wafer device. (b,c) Optical image and corresponding X-ray image of the hardware tools obtained by the Cs_3_Cu_2_I_5_ wafer detector, the dose rate for imaging is (b) 209 μGy_air_ s^−1^ and (c) 10.05 μGy_air_ s^−1^, respectively.

**Theoretical sensitivity (*S*_0_, the case without gain) calculation of the X-ray detector:**

The theoretical sensitivity *S*_0_ can be calculated by the following equation: ^[1]^

$$S_{0}= \frac{\frac{\phi}{X}\bar{E}\beta}{W_{\pm}}e\eta$$

where $W_{\pm}$is the ionization energy, $\frac{\phi}{X}$the number of photons per unit of exposure, $\bar{E}$is the mean energy of the X-ray photons, $\beta$ the energy absorption efficiency of X-rays, *e* the elemental electron charge, and *η* the charge collection efficiency. For general comparison, the $W_{\pm}$ is calculated by the empirical model as: $W_{\pm}$= 2*E_g_* + 1.43 eV.^[2]^ The $\bar{E}$of the X-ray is ~ 40 keV for the X-ray source used in this work. According to previous reports,^[3]^ $\frac{\phi}{X}$ is about 210 747 photons mm^-2^ mR^-1^, since 1 mR = 8.76×10^-6^ Gy, $\frac{\phi}{X}$ equals to 2.4 × 10^12^ photons Gy_air_ ^-1^cm^-2^. The *β* and *η* is assumed to be 100% to obtain the theoretical detection sensitivity. Consequently, the *S_0_* (the case without gain) of Cs_3_Cu_2_I_5_, BA_2_PbBr_4_, PA_2_PbBr_4_, and PEA_2_PbBr_4_ wafer detectors is 1.12 $\times$10^3^ $\mu$C Gy_air_ ^-1^ cm^-2^, 1.28 $\times$10^3^ $\mu$C Gy_air_ ^-1^ cm^-2^, 1.3$\times$10^3^ $\mu$C Gy_air_ ^-1^ cm^-2^, and 1.33$\times$10^3^ $\mu$C Gy_air_ ^-1^ cm^-2^, respectively.

**References**

[1] Y. Liu, Y. Zhang, X. Zhu, J. Feng, I. Spanopoulos, W. Ke, Y. He, X. Ren, Y. Zhou, F. Xiao, K. Zhao, M. Kanatzidis, S. Liu, *Adv. Mater.* **2021**, 33, 2006010.

[2] R. Devanathan, L. R. Corrales, F. Gao, W. J. Weber, *Nucl. Instrum. Methods Phys. Res. Sect. A* **2006**, 565, 637-649.

[3] W. Pan, H. Wu, J. Luo, Z. Deng, C. Ge, C. Chen, X. Jiang, W.-J. Yin, G. Niu, L. Zhu, L. Yin, Y. Zhou, Q. Xie, X. Ke, M. Sui, J. Tang, *Nat. Photonics* **2017**, 11, 726-732.
